# Predicting coastal erosion susceptibility in Bangladesh under climate scenario via machine learning techniques

**DOI:** 10.1371/journal.pone.0334347

**Published:** 2025-11-05

**Authors:** Sakib Hosan, Sondipon Dey Pranta, Tahdia Tahmid, Mafrid Haydar, Al Hossain Rafi

**Affiliations:** Department of Urban and Regional Planning, Khulna University of Engineering & Technology (KUET), Khulna, Bangladesh; Sathyambama Institute of Science and Technology: Sathyabama Institute of Science and Technology (Deemed to be University), INDIA

## Abstract

Using advanced machine learning methods along with geospatial data and climate estimates, this study found areas in Bangladesh that are likely to experience coastal erosion. Twenty important factors were looked at, such as meteorological, geographical, hydrological, tropological, and land-use variables. The normalized difference vegetation index (NDVI) was found to be the most important factor. An ensemble machine learning technique was used to figure out how susceptible coastal areas are to erosion. Several types of boosting techniques were used, including Extreme Gradient Boosting (XGBoost), Light Gradient Boosting Machine (LightGBM), Categorical Boosting (CatBoost), Gradient Boosted Decision Trees (GBDT), and AdaBoost. Random Forest, Decision Tree, Treebag, Bagging, and Averaged Neural Network (avNNet) were also used. The area under the curve (AUC) and receiver operating characteristic (ROC) values were used to check how well the model worked. XGBoost had the best AUC, at 0.95, which means it did a very good job of classifying places that are likely to be washed away by erosion. The study of geography showed that most of the models showed moderate-risk areas, which made up 71.82% to 79.36% of the whole area. Some of the districts that were identified as mostly high-risk were Bhola (19.41%), Cox’s Bazar (26.20%), and Patuakhali (21.47%). A lot of low-risk zones were found in places like Jashore and Narail, on the other hand. Predictions of how likely erosion will be in the future based on different warming models to make Representative Concentration Pathways (RCPs 2.6, 4.5, 6.0, and 8.5) for the years 2040, 2060, 2080, and 2100, we used data from the Coupled Model Intercomparison Project (CMIP5). In line with RCP 8.5, the number of high-risk places is expected to rise to 50% by 2080 and to 40% by 2100. RCP 6.0 had a smooth shift, and in high-risk areas, there were only small rises. RCP 2.6, 4.5, and 6.0 all went up a little. These results show that Bangladesh’s shore is becoming more likely to be worn away by erosion as temperatures rise. They stress how important it is to quickly react, handle coastal areas, and use planning methods that are resilient to climate change.

## Introduction

As waves continuously alter the morphology of the shore over time, coastal erosion gradually erodes away the land. The high rate of coastal erosion significantly raises coastal susceptibility at the national, regional, and international levels in addition to other coastal disasters such as tidal surge, cyclones, and flooding [1,2]. For people who live in coastal, deltaic, and riverine systems worldwide, erosion poses serious environmental problems [3–6]. It is typically the result of both natural and anthropogenic forces, which may work alone or in concert [7,8]. Anthropogenic factors, including building ports, coastal barriers, groins, waterways, tourist hotels, gardens, and dredging inlets, as well as natural ones like the wind, surges, currents, particles, active tectonics, and sea level rise, all contribute to coastal erosion [9]. The shape of the coasts is permanently altered when the sediment is removed from the sediment sharing system [10]. Despite the fact that no part of the planet is immune to environmental dangers, certain places are geographically more susceptible to their impacts than others. For instance, the low-lying coastal areas are extremely susceptible due to the effects of climate change [11]. Although the effects of coastal erosion are limited to coastal regions, these regions are home to more than 40% of the global population and a diverse spectrum of coastal ecosystems that offer a multitude of services [12]. The coastal areas of Bangladesh are no exception to this reality, as they cover 32% of the terrestrial area of Bangladesh and are home to 26% of the total population of the country [13]. Bangladesh loses 34 square kilometers of land per year, and this loss could get worse as sea levels rise [14]. Coastal regions, especially mangrove forests, are prime candidates for protection [15]. Along the coast, loss of land is common, displacing people, disrupting livelihoods, and imperiling valuable ecosystems like the Sundarbans, the world’s largest mangrove forest. Coastal resilience is one of the priority sustainable development issues of Bangladesh because of intensifying cyclones, increased salinity intrusion, and unpredictable weather patterns caused by climate change.

Various techniques have been used by the researchers, which include expert-based methods and real-time data analysis. As alternative methods for the prediction of beach morpho dynamics, different machine learning methods have been developed and tested over the last several years. In some studies, researcher have trained and validated models such as the Functional Trees (FT), Multi-Layer Perceptron (MLP), Naïve Bayes (NB), Support Vector Machines (SVM), Logistic Regression (LR) and Deep Learning (DL) with the physical and environmental parameters such as wave height and direction, horizontal flow magnitude, geology, and slope, along with coastal erosion inventories [16–18]. The Random Forest (RF), Gradient Boosted Regression Tree (GBRT), and Tree Ensemble (TE) machine learning models were used by [19–23] to identify the zones of maximum gully erosion (GES). Some other studies [24–28] focused on three deep learning techniques: Convolutional Neural Network (CNN), Recurrent Neural Network (RNN), and Long-Short Term Memory (LSTM) for spatial forecasting. In research by Naceur et al. [29], outlined the N’fis River Basin in Morocco’s gully erosion using RF, AdaBoost, and GBDT models. With an AUC of 0.932, RF was the top-performing model, followed by GBDT (0.893) and AdaBoost (0.902). Eloudi et al. [20] likened six models in mapping the susceptibility of gully erosion: AdaBoost, XGBoost, GBM, TreeBag, RF, and C5.0. With an AUC of 90.80%, the C5.0 model performed optimally, followed closely by RF (90.10%), XGBoost, and AdaBoost (both 90%).

A number of studies have used traditional geospatial and statistical techniques to investigate erosion patterns and suggest conservation measures in Bangladesh. The Revised Universal Soil Loss Equation (RUSLE) with GIS has been used in a number of studies to predict soil erosion and detect erosion hotspots. Using the Global Soil Erosion Modeling Platform (GloSEM) Rahman et al. and Islam et al. [30,31] measured erosion rates and validated their findings. Despite yielding valuable information, these studies employed deterministic models assuming rigid links between erosion rates and input variables. This works against adaptive erosion management and reduces their potential to forecast future erosion scenarios in the face of changing climatic conditions. Socio-economic erosion research pays greater attention to adaptation and resilience compared to predictive modeling. Hasan et al. [32] used K-Nearest Neighbors (KNN), XGBoost, and Random Forest (RF) were utilized to evaluate flood risk in the coastal areas of Bangladesh. RF was the most accurate with an accuracy of 86.7%, followed closely by XGBoost (86.3%). Barguna, Bhola, and Patuakhali districts were found to be particularly susceptible by the research. While Mamun et al. and Islam et. al [31,33] took into account coping mechanisms and communal susceptibilities, they didn’t involve high-resolution spatial modeling for future hazard forecasting. However, there are a number of issues with existing approaches because deterministic models like RUSLE and GloSEM are based on static assumptions; they are unable to capture dynamic drivers like changing land use, extreme weather, and shifting climatic trends. Moreover, these models do not incorporate real-time data, thus limiting their use in adaptive erosion management. For better predictive accuracy, future research should emphasize the creation of hybrid models that combine machine learning and geographic methods. Furthermore, an integrated approach that combines socioeconomic risk analysis with physical erosion modeling in support of sustainable land management would provide holistic insight.

Thus, the main goal of this research is to utilize an exhaustive set of machine learning (ML) models to evaluate the geographical aspects of coastal erosion in Bangladesh’s dynamic coastline by combining geological, climatic, hydrological, topographical, and human-induced parameters. This study aims to: (i) identify and rank the most important drivers controlling the susceptibility of coastal erosion; (ii) compare the predictive capabilities of different individual and ensemble-based machine learning models for forecasting erosion-susceptible zones; (iii) generate high-resolution spatial susceptibility maps for the current scenario. The results aim to inform planners, environmental managers, and policymakers to implement well-informed, spatially explicit plans for the sustainable management of coastal zones in the face of present and future climate change.

## Materials and methods

### Overview of the study location

**Fig 1:**
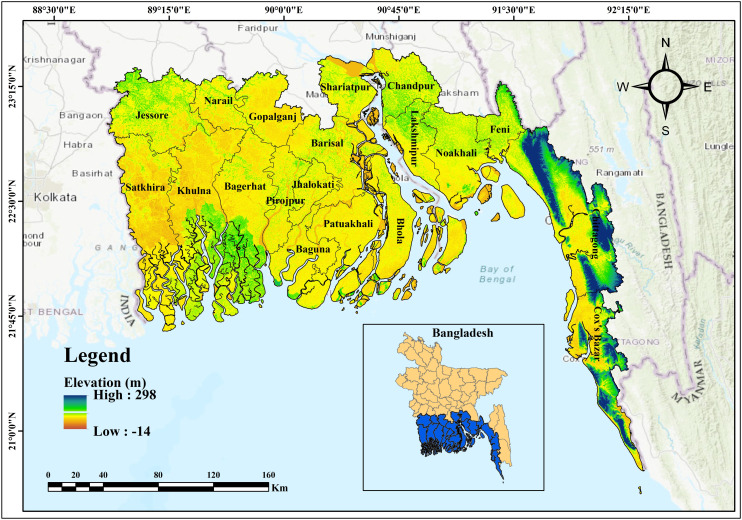
Study area map of the coastal region of Bangladesh.

The research area (in Fig 1) is situated in the coastal region of Bangladesh, in the southern part of the country, lying between longitudes 80° 0’ E to 91° 0’ E and latitudes 21° 30’ N to 22° 30’ N [34]. The coastal region of Bangladesh covers 47,200 km², including land, islands, and several water bodies, constituting around 32% of the nation’s total area [35]. It is subdivided between the interior coast (23,265 km²) and the exposed coast (23,935 km²), with the latter being directly influenced by tidal surges and cyclonic activity from the Bay of Bengal [36]. The coastal zone of the nation is divided into three zones: western, central, and eastern, based on geomorphological characteristics, spanning approximately 27,150 km², 12,040 km², and 8,010 km² of coastal land area, respectively [37]. The eastern coast of Bangladesh is characterized by lofty, hilly land with urbanization and intricate flood dynamics; the middle portion is low-lying and extremely vulnerable to tidal and river floods; whereas the western region is covered with mangrove forests and dynamic delta landscape that is prone to erosion and saltwater inundation [35,37,38]. The study aims at the coastal belt of Bangladesh, where erosion threatens human habitation and natural ecosystems significantly [30]. The region’s susceptibility to tidal surges, storm effects, and sea level rise at high levels renders it a significant place for assessing erosion risk [39]. This study aims to enhance the predictive accuracy and establish a sustainable coastal erosion mitigation framework in Bangladesh through the application of advanced machine learning algorithms and GIS-based susceptibility modeling. The research employs high-resolution geographical data and climate change scenarios to more accurately identify locations susceptible to erosion. This integrative approach safeguards Susceptibility ecosystems and societies on the coast of Bangladesh by creating a scientific basis to formulate sustainable coastal management policies and long-term adaptation strategies, while facilitating evidence-based decision-making.

### Description of data

The model parameters were chosen and identified following a comprehensive review of the literature. Twenty factors mentioned in Table 1 are employed for reducing coastal erosion in Bangladesh. Digital Elevation Models (DEMs) help pinpoint low-lying coastal regions susceptible to sea-level rise, tidal inundation, and wave erosion, where low-lying places are more Susceptibility to erosion because of their closeness to water and coastal dynamics, whereas elevated regions are safeguarded [24,40–43]. The Shuttle Radar Topography Mission (SRTM) Digital Elevation Model (DEM) data are used for spatial analysis. The gradient of the terrain or slope influences hydrological and geomorphological processes [44]. The coastal slope influences surface runoff, sediment transport, and stability, and on the Senegal coast, gentle slopes are more susceptible to erosion due to inadequate water drainage and surface water accumulation, whereas steeper slopes may mitigate wave impact but heighten the risk of landslides [24,42,43,45]. The slope layer was generated from the DEM. Vertical Distance from Channel Network (VDCN) quantifies elevation of a location with adjacent drainage channels [46]. It indicates the accumulation of surface water and fluvial activity, where VDCN values that are lower show a higher risk of erosion from overland flow and waterlogging, and those that are increasing indicate a reduced risk [43]. DEM made it possible to derive VDCN. The Topographic Wetness Index (TWI) evaluates local slope and upstream contributing area in order to measure soil texture distribution [47]. High TWI values indicate water accumulation and saturation, which can compromise soil stability and enhance susceptibility to erosion [43]. Conversely, low TWI values depict dry, more stable conditions. The DEM was utilized to derive the TWI via hydrological modelling. The Topographic Position Index (TPI) quantifies the height of a point above or below the average height of its surroundings [48]. This index can be utilized to classify hills, valleys, and plains. Positive values of TPI express high landforms with lower susceptibility to erosion, whereas negative values of TPI signify depressions and valleys with high water retention capacity [43,49]. The TPI layer was developed based on the 30-meter resolution DEM data. The Topographic Ruggedness Index (TRI) quantifies height variation in a region to evaluate terrain heterogeneity [50]. Cliffs and dunes have elevated TRI values, which can either exacerbate or mitigate erosion based on substrate hardness where Rugged terrain with pliable substrates may erode more rapidly, whereas rocky regions may exhibit greater resistance [43,51]. TRI was derived from DEM. The flow of water over landforms is influenced by the curvature of the ground [52]. Concave landforms retain water and silt, heightening erosion risk, whereas convex parts facilitate drainage and diminish moisture retention [24,43,53]. The curvature layer was generated using GIS Curvature with DEM data. Precipitation and potential evapotranspiration dictate climate aridity in the Aridity Index [54]. An increased aridity index indicates a drier environment with diminished vegetation, rendering land more Susceptibility to wind and water erosion, whereas low-aridity regions possess abundant flora, which stabilizes the soil [43,55]. This index was developed using WorldClim datasets. Microclimatic elements such as wind patterns and sunlight exposure are influenced by slope aspect [56]. Coastal windward slopes are more susceptible to wave and wind forces, heightening the risk of erosion, whereas leeward slopes are protected [43]. DEM data yielded aspect values. The Normalized Difference Vegetation Index (NDVI) is a widely utilized remote sensing statistic for assessing vegetation density and health [57,58]. Elevated NDVI values indicate abundant vegetation, enhancing soil structure and mitigating erosion, and low NDVI signifies diminished vegetation or bare soil, which is more susceptible to erosion [24,43]. NDVI data were obtained from Sentinel-2 imagery at a resolution of 10 meters. Precipitation influences the hydrological cycle and erosion, where heavy rainfall enhances surface runoff and soil saturation, accelerating erosion [43,59]. CHIPS precipitation data were utilized in the study. The mean wind speed significantly influences coastal erosion by producing wave energy and transferring aeolian silt [60]. Wind velocity amplifies wave dynamics, resulting in coastal erosion and the inland transport of materials so calm circumstances diminish coastal erosion [43,61]. Wind speed data was used from ERA5 reanalysis datasets in order to examine these effects. Additionally, geomorphology examines landform classifications and their geological characteristics, which influence coastal erosion [62]. Rocky coastal formations such as cliffs and headlands exhibit superior erosion resistance compared to sandy beaches [42,63]. The geomorphological categories utilized in this study were gained from the Geological Survey of Bangladesh. Soil texture effect coastal erosion where Clay soils exhibit greater compaction and erosion resistance compared to sandy soils, which are readily moved by wind and water [42,64]. Soil texture data was obtained from the FAO Global Soil Map. The distance to the coastline quantifies the proximity of a location to the shore that influence coastal erosion [42]. Coastal regions are especially susceptible to wave dynamics, tidal influences, and saline intrusion, which intensify erosion [39]. Distance was ascertained via shape file data. The spatial distribution of anthropogenic and natural surface elements that influence coastal erosion, such as urban areas, forests, and agricultural lands, is depicted by Land Use and Land Cover (LULC) statistics [15,24,42]. The research employs Esri’s Sentinel-2 Global Land Cover 2023 datasets at a 10-meter resolution, a globally recognized dataset derived from high-resolution satellite imagery for land use and land cover analysis [65,66]. The distance to a river indicates a location’s closeness to fluvial systems that provide water and sediment that affect coastal erosion, where it occurs more frequently in proximity to rivers than at greater distances [67]. The river distance layer employs humanitarian vector datasets. Fault lines can influence ground stability and long-term erosion, where active tectonic faults may experience subsidence or uplift, hence augmenting coastal instability and sediment redistribution [16]. Stable locations are typically situated distant from fault lines. Increasing mean sea level, a long-term average of sea surface elevation, induces coastal erosion [39]. Elevated sea levels exacerbate coastal erosion, saline intrusion, and storm surges. Stable or declining sea levels mitigate these effects. The Permanent Service for Mean Sea Level utilized MSL data. The erosion resistance of an area is contingent upon its geological materials. Basalt and granite exhibit durability, but shale and sedimentary formations are more susceptible to erosion under hydrodynamic forces [68]. From the USGS Earth Explorer, utilized geological data.

**Table 1:** Data sources of parameters for mitigating coastal.

Category	Indicators	Data Sources	Data Type	Unit	Resolution	Period
Topographical	Aspect	Derived from DEM	Raster	–	30m	2024
	Curvature	Derived from DEM	Raster	–	30m	2024
	Digital Elevation Model	SRTM	Raster	Meter	30m	2024
	Distance to Coastline	Author	Vector	Degrees (°)	–	2024
	Slope	Derived from DEM	Raster	Degrees (°)	30m	2024
	Topographic Position Index	Derived from DEM	Raster	–	30m	2024
	Topographic Ruggedness Index	Derived from DEM	Raster	–	30m	2024
Hydrological	Distance to River	Humanitarian Datasets	Vector	Degrees (°)	–	2024
	Mean Sea Level	Permanent Service for Mean Sea Level (PSMSL)	Vector	Meter	–	–
	Topographic Wetness Index	Derived from DEM	Raster	–	30m	2024
	Vertical Distance from Channel Network	Derived from DEM	Raster	–	30m	2024
Meteorological	Aridity Index	WorldClim	Raster	–	30m	2024
	Mean Wind Speed	ERA5	Raster	km/h	30m	2024
	Precipitation	CHIPS	Raster	mm	30m	2024
Geological	Distance to Faultline	Author	Vector	Degrees (°)	–	2025
	Geology	USGS Earth Explorer	Vector	–	–	2024
	Geomorphology	Geological Survey of Bangladesh	Raster	–	30m	2024
	Soil Texture	FAO Global Soil Map	Vector	–	–	2024
Land Use & Human Activity	Land Use and Land Cover	Sentinal-2 Image	Raster	–	10m	2024
	Normalized Difference Vegetation Index	Sentinal-2 Image	Raster	–	10m	2024

### Erosion inventory mapping

**Fig 2:**
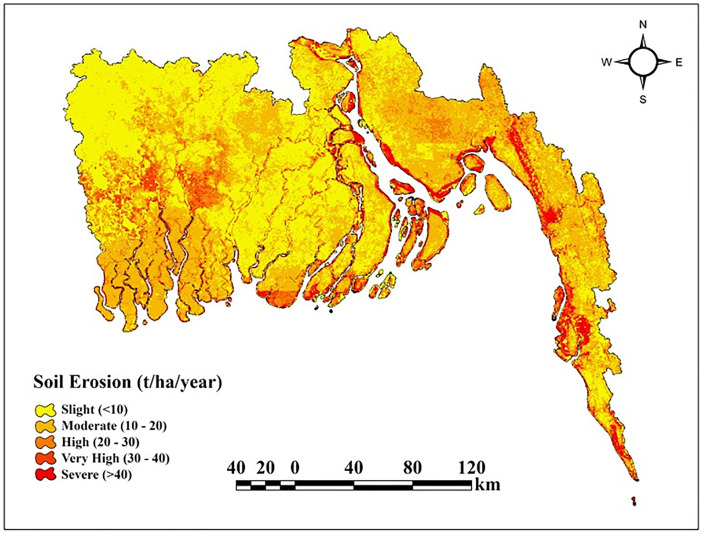
Geospatial Distribution of Coastal Erosion.

Fig 2 illustrates the coastal erosion map, demonstrating the spatial distribution of soil erosion intensity throughout the research area. This map was produced utilizing the Revised Universal Soil Loss Equation (RUSLE) model, which quantifies yearly soil loss in tons per hectare per year. The Revised Universal Soil Loss Equation (RUSLE) was developed by Renard [69] and is widely used and popular all over the world for average annual soil loss estimation. [70]. In this research, the model was used to analyze the potential soil erosion in the coastal zone of Bangladesh. As [71], said, the RUSLE model is represented by the equation A = R × K × LS × C × P, where A is the potential yearly soil loss in tons per hectare per year, R is the rainfall-runoff erosivity factor in megajoules per hectare per hour per year, K is the soil erodibility factor in tons per meter per hour, LS is the slope length and steepness factor, C is the cover and management factor, and P is the conservation support practice factor. CHIRPS (Climate Hazards Group InfraRed Precipitation with Station Data) daily precipitation records were utilized in the computation of the rainfall erosivity factor, R [72]. The K factor for soil erodibility was computed from data on several soil types where categorical reclassification was performed based on soil texture classes, and empirically estimated K values were allocated to each class [73]. The Topographic Factor (LS) is the compound effect of slope steepness and length, whereby the slope calculation was performed based on the Shuttle Radar Topography Mission (SRTM) data and the digital elevation model (DEM). Once the slope had been converted from degrees to percentages, the LS was then calculated once the conversion had been carried out. The Cover Management Factor (C) was derived by calculating the Normalized Difference Vegetation Index (NDVI) from the Landsat-8 photo surface reflectance bands [74]. A function of NDVI was applied to transform vegetation density to a C factor. This correction normalizes the values of C to between 0 and 1 and implies that areas with denser plant cover are less susceptible to erosion. Slope data and MODIS land cover type (LC_Type1) were utilized in developing the Support Practice Factor (P) [75]. Diverse P values were assigned to the distinct land cover types and slope categories, grounded in the methodologies defined in the literature. The application of effective soil conservation techniques resulted in reduced P values on gentle slopes of agricultural land. The final soil loss map was generated by multiplying the five RUSLE components (R, K, LS, C, and P) on a pixel-by-pixel basis. This yielded estimated yearly soil loss figures articulated in tons per acre. Following the estimation of annual soil loss in tons per hectare per year (t ha ⁻ ¹ year ⁻ ¹) over the study area, the results were visualized and categorized into two classes of erosion severity, employing a threshold-based classification method to establish the coastal erosion inventory. Based on [76–79], all pixels exhibiting soil loss rates exceeding 30 t ha ⁻ ¹ year ⁻ ¹ were classified as erosion-prone locations and assigned a rating of 1 whereas, all other pixels with values equal to or below this threshold were classified as non-erosion zones and awarded a value of 0. This inventory not only offers reliable erosion occurrence data grounded in quantitative soil loss estimates but also acts as a fundamental input for further susceptibility modeling and spatial analysis.

### Selection of explanatory variables

Multicollinearity, a prevalent problem in multiple regression, occurs when independent variables exhibit significant correlation, which may inflate the standard errors of coefficients and diminish statistical reliability [80]. Pearson correlation matrices provide independent variable correlation coefficients to discover multicollinearity. Multicollinearity is possible if two variables have a high correlation near 1 or −1 [81]. However, Variance Inflation Factor (VIF) and Pearson’s correlation coefficient (p-value) are employed to measure multicollinearity more precisely [80]. A VIF below 10 shows a significant correlation that may pose issues, while a VIF over 10 signifies severe multicollinearity, potentially distorting regression estimates [82].

### Analytic methods, including boosting and bagging technique

**Fig 3:**
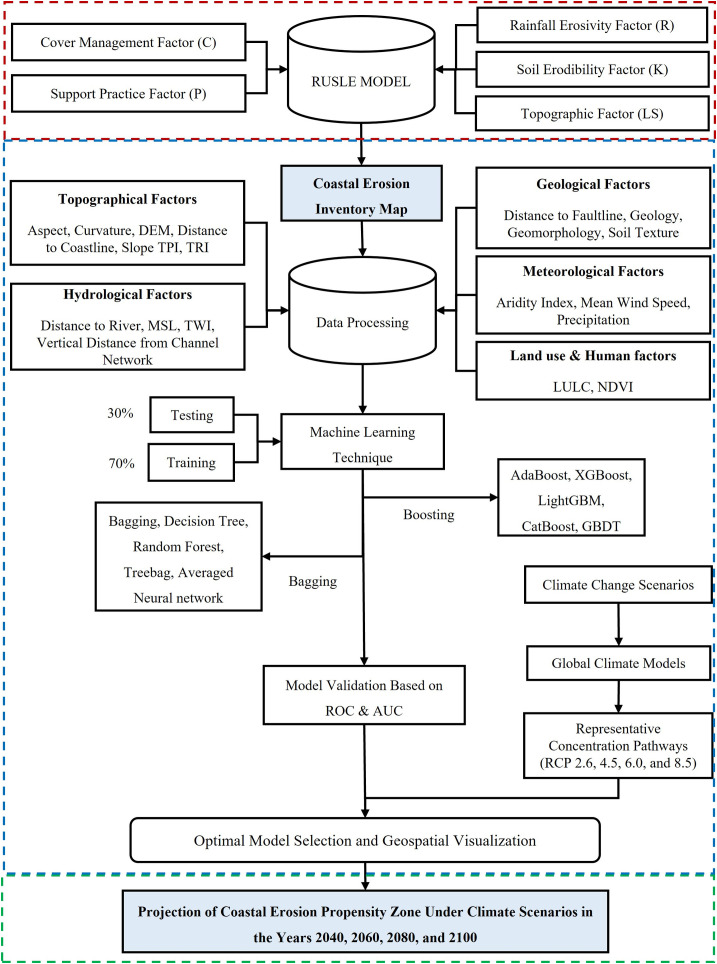
Methodological framework.

This study employes five boosting and five bagging algorithms to predict coastal erosion in Bangladesh (Fig 3). XGBoost is one of the strong and robust methods that is utilized in the machine learning field is referred to as [83–85]. For performing user-defined predictive tasks such as regression, classification, ranking, and supervised learning, XGBoost is a flexible and efficient ensemble learning framework that makes use of gradient boosting and tree topologies [86,87]. The dataset was normalized and divided into training and testing subsets using stratified sampling. Sample weights were utilized throughout training to address class imbalance. XGBoost classifier was trained with 100 trees (n_estimators = 100), a maximum depth of 5, and a learning rate of 0.03, utilizing complete subsampling and feature utilization (subsample = 1, colsample_bytree = 1). Robust L1 and L2 regularization (reg_alpha = 8, reg_lambda = 8) was employed to mitigate overfitting. In the current study, the XGBoost algorithm was used, and seventy percent of the database was allocated to training the model while thirty percent was allocated to testing the model.

AdaBoost employs adaptive boosting for classification trees [88]. Adaboost improves the classification precision of machine learning based on classification, and the non-parametric method efficiently identifies outliers without the need to identify bad learners [88,89]. AdaBoost training starts with the creation of a base decision tree through the equal weighting of the dataset [89,90]. AdaBoost is susceptible to noisy or corrupted data [91]. AdaBoost classifier was executed utilizing a decision stump (a decision tree with a maximum depth of one) as the foundational learner. The model was trained using 100 estimators and a learning rate of 0.03. The issue of class imbalance in the training data was mitigated by utilizing sample weights derived from balanced class weighting. The authors employed the AdaBoost model with a 70% and 30% training to test split.

Gradient Boosting Decision Trees, simply known as GBDT, is a sophisticated combination algorithm that iteratively enhances regression decision trees to reduce classification errors at each step along the process [92,93]. Numerous weak classifiers are constructed across different iterations, and learning rates are smaller, so more iterations need to be conducted to secure the same fit [94]. It is the correct tree algorithm on which the GBDT depends that scales up the loss function with the negative gradient as a form of residuals, and whose result results in the gradual diminution of residual values [95,96]. Because of its use of many weak decision trees, GBDT offers stability and accurate projections. Gradient Boosting classifier was trained with 100 estimators, a maximum tree depth of 5, a learning rate of 0.03, and subsample and feature sampling rates of 0.8 to enhance generalization. It is thus the best solution. 70% of the time is spent in training and 30% in testing here.

LightGBM is an open-source gradient boosting library founded by Microsoft that utilizes the histogram-based method to optimize training efficiency and reduce computational complexity [97,98]. In addition, it has enhanced network communication to enable additional parallel learning, for example, the parallel decision tree voting technique and grows trees by managing variance gain and sorting training examples in decreasing order based on absolute gradient values [99,100]. The LightGBM classifier was trained using 100 estimators, a maximum tree depth of 5, a learning rate of 0.03, and complete subsampling of both data and features (subsample = 1, colsample_bytree = 1). Robust regularization was implemented through L1 (reg_alpha = 8) and L2 (reg_lambda = 8) penalties, while class imbalance was mitigated by employing balanced class weights. Feature significance was derived and standardized to ascertain the most significant predictors. Thirty percent of the data was used for testing, and seventy percent was used for training for this study.

CatBoost is a very advanced machine learning method that has been effective in capturing a variety of factors influencing water potential as well as properly handling noisy and heterogeneous information [101]. As a Gradient Boosting Decision Tree-based technique, it is particularly well-suited to model complex relationships and heterogeneous feature sets [101,102]. Sample weights were calculated to address class imbalance, with class weights explicitly designated according to the imbalance ratio. A CatBoost classifier was trained with 100 iterations, a maximum depth of 5, a learning rate of 0.03, and an L2 regularization value of 10. Feature subsampling was established at 70% (rsm = 0.7) to improve generalization. According to this study, a statistically target-oriented approach is employed wherein seventy percent of the training data is randomly chosen, and thirty percent is reserved for testing.

Random Forest (RF) is a statistical ensemble machine learning method employed most of the time to forecast the target variable [24,103,104]. The method employs bootstrapping techniques along with stochastic binary trees for the purpose of using some portion of the data that provides a better representation. The model is constructed by the random selection of training from the initial set of observations [105]. The bagging classifier was trained with 100 decision trees, each with a maximum depth of 5, and minimum sample split and leaf sizes adjusted to mitigate overfitting. Probabilities were computed for predictions, and feature importance was averaged and normalized across all base estimators to find significant contributors. Within the experiment, the information is divided into two sets: thirty percent is held out for testing, and seventy percent for training.

The bagging strategy for an ensemble entails repeatedly training the same algorithm on numerous distinct subsets of the training set [105,106]. The average of all the projections from the sub-models forms the basis of the final forecast. This process minimizes heterogeneity in classification, which ultimately increases overall accuracy [107]. Bagging is an approach that can be used to improve accuracy in situations where big fluctuations in the training set significantly affect the subsequent prediction, where the outcome of a test sample is determined through the vote of the ensemble that received the highest vote [108,109]. A Bagging Regressor consisting of 100 decision trees, each with a maximum depth of 5, was trained, utilizing 1% of the training data per tree to mitigate overfitting while employing all features. Despite being intended for regression, the model’s continuous predictions were utilized as probabilities for binary classification. The significance of features was averaged throughout the ensemble and normalized to emphasize principal predictors. Two data sets were established for the study, thirty percent of which were reserved for testing and seventy percent reserved for training.

In order to execute the decision tree (DT) method, the data are first split into various regions according to the independent variables, and rules for each region are subsequently developed [110,111]. The primary application of decision tree models is to obtain the development of a model for predicting results on the basis of input characteristics. Decision trees are significantly effective in classification, regression, and prediction tasks [112,113]. This code trains a Decision Tree classifier on the specified dataset, utilizing features associated with environmental and geographic indices to predict a binary target variable. The data is divided into training and test sets based on the DT strategy, with thirty percent of the data left for testing and seventy percent left for training.

Treebag is an ensemble learning method that takes advantage of the benefits of decision trees and bagging in order to improve prediction accuracy that is achieved when multiple decision trees are made by constructing bootstrapped subsets of the training data, each of which was trained separately [114,115]. Through the mean of error averaging over many trees, Treebag improves the stability and robustness of decision tree implementations and this technology has been applied with high frequency to vast topics, including issues of classification and regression, and has achieved better performance than single decision tree models [116–119]. With a thirty percent test split and seventy percent train split, A Treebag classifier with limited complexity (maximum depth of 5, minimum samples for split and leaf) was employed to mitigate overfitting. The Bagging Classifier was subsequently trained using 100 estimators, each utilizing the complete sample and feature set, while employing parallel processing for enhanced efficiency. Treebag is utilized in the present research.

Averaged Neural Network, or avNNet, is an ensemble learning method utilized for increasing predictive accuracy by averaging the output of numerous neural networks [120]. It is a method that does not rely on one trained network but one that utilizes predictions of multiple models trained independently and then averages the models’ output [121]. When overfitting problems must be resolved and model robustness has to be enhanced for hard learning tasks, such a method is quite helpful. Through the utilization of various neural networks trained on various subsets of the data or initially charged with various weights, avNNet has been found to enhance classification and regression task stability, as demonstrated by research [122,123]. This renders the process of averaging to be a stable method to employ in machine learning settings since it reduces the impact of individual network biases and improves the accuracy of the entire output [124]. The avNNet classifier was executed with a multilayer perceptron architecture featuring two hidden layers containing 100 and 50 neurons, respectively. The avNNet technique is employed within this study by splitting 70% of the dataset for training purposes and 30% for testing purposes, according to the dataset.

For all models, to rectify class imbalance, we utilized the class_weight = ‘balanced’ argument, which modifies weights according to class frequencies. Moreover, stratified sampling was employed in the train-test division to maintain class distribution.

### Model validation

Better performance in machine learning models is associated with greater AUC values [125]. The study graphs ROC curves for all of the models through software in the programming language Python. The ROC curve is a plot of a classifier’s performance in distinguishing between numerous classes [126,127]. The Area Under the Curve (AUC) is a scalar measure of the model’s performance that originated from the Receiver Operating Characteristic (ROC) curve. The data was divided in a training-to-testing proportion of 70% and 30%, so that there was an equilibrium between model training and validation. The AUC score and ROC curve were utilized as primary measures of performance, with significantly larger AUC scores indicating superior classification capacity (Table 2). The best classifier will have a ROC curve that is clumped in the upper-left quadrant, which indicates that it has a high level of both sensitivity and specificity [86,126,127]. According to the set standards, values of area under the curve (AUC) range between 0 and 1, and high values indicate improved model discrimination capacities by a greater margin.

**Table 2:** Classification of Model Performance According to AUC (Area Under the Curve). Values [86,126,127].

AUC Range	Classification	Interpretation
0.91–1.00	Excellent	Very high classification accuracy, close to perfect performance
0.81–0.90	Very Good	Strong predictive ability with a low misclassification rate
0.71–0.80	Good	Reliable model performance, though not flawless
0.61–0.70	Satisfactory	Significant predictive efficacy, acceptable for general use
0.51–0.60	Unsatisfactory	Slightly better than random chance; needs further improvement

A confusion matrix evaluates the effectiveness of a classification model by displaying true positives (nodes accurately identified as positive), true negatives (nodes accurately identified as negative), false positives (nodes inaccurately identified as positive), and false negatives (nodes inaccurately identified as negative), thereby providing a thorough assessment of the model’s performance across different classes [128,129]. This matrix enables the assessment of model correctness, the detection of misclassifications, and the calculation of critical metrics such as the F1-score, which improves model performance. Accuracy represents the proportion of correct forecasts to total predictions, with increased accuracy indicating greater overall correctness; Precision measures the ratio of true positives to total predicted positives, with high precision signifying fewer false positives; recall evaluates the ratio of true positives to total actual positives, where high recall reduces false negatives; the F1-score integrates precision and recall as their harmonic mean, making it beneficial for assessing models, especially in imbalanced datasets [129].

### Future climate change models

Forecasts of impending climate change are required for the description of mechanisms of coastal erosion, for making estimates of potential danger to coastal residents, and guiding the development of effective adaptation measures and mitigations. The fifth Coupled Model Intercomparison Project (CMIP5) phase will provide a cutting-edge multimodel dataset that will advance our knowledge of climate variability and climate change [130]. The framework provides an excellent platform for climate variability assessment and its effects on water resources [131,132]. Representative Concentration Paths (RCPs) establish several greenhouse gas concentration pathways, such as a set of four new pathways created for the use of the climate modelling community as a basis for long-term and short-term modelling studies [133]. These RCPs serve as the basis for CMIP5 based projections wherein RCP 2.5, RCP 4.5, RCP 6.0, and RCP 8.5 represent the four RCP scenarios applied in this study. The purpose of this study is to evaluate future climate conditions and their implications that may occur [134]. Particularly, the research assesses the sea level average pressure under various scenarios using projection data for 2040, 2060, 2080, and 2100. Sea level rise causes the acceleration of the coastal erosion process by intensifying the frequency of wave impact on the coastlines, especially during storms and periods of high tides [135]. This results in the shoreline erosion process happening frequently and more intensely [136]. In this study, climate projection data were retrieved from the website of the NCAR GIS Initiative Climate Change Scenario (https://gisclimatechange.ucar.edu), where projections of climate are provided for public access.

### Future coastal erosion projection

A global climate model simulated projected coastal erosion risk zone maps for 2040, 2060, 2080, and 2100 using multiple climate change scenarios defined based on Representative Concentration Pathways (RCPs). A raster calculator was used to compute a spatial correlation between simulated coastal erosion effects and projected coastal erosion using outputs of a boosting model, i.e., XGBoost. The risk maps produced were then systematically put into various categories from low to highly vulnerable to coastal erosion. These projections provide useful insight into the expected increase in coastal vulnerability in Bangladesh and are a critical input to policymakers for formulating long-term adaptive shore protection and general disaster risk reduction strategies.

## Results and discussion

### Description of topographical factors

**Fig 4:**
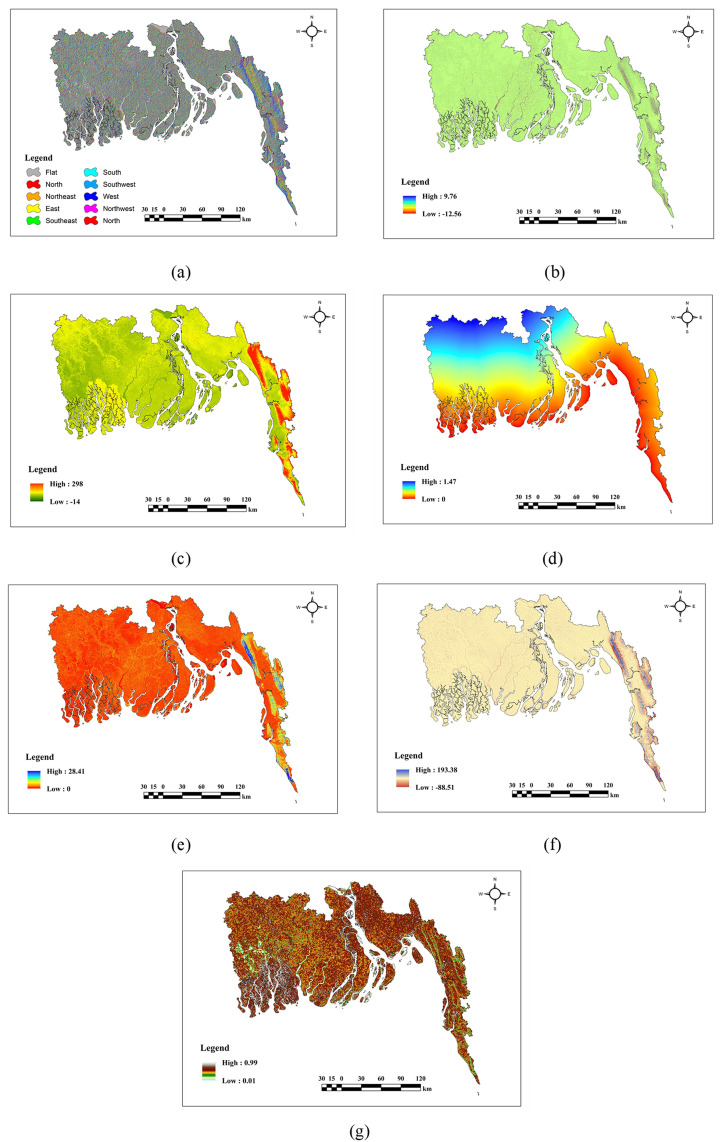
Topographical factors (a) Aspect, (b) Curvature, (c) DEM, (d) DtC, (e) Slope, (f) TPI, (g) TRI.

The topographical factors in Fig 4 exhibit clear spatial changes throughout the region. Aspect(a) and Curvature(b) exhibit a mixed pattern with no distinct predominance in any specific area. DEM(c), Slope(e), and TPI(f) demonstrate elevated values in the northern and eastern highland regions, whereas lower values are found in the southern and western lowlands, with intermediate values distributed throughout the center sections. DtC(d) has lowered values predominantly in the southern regions, progressively escalating toward the north. TRI(g) exhibits higher levels in the central and northeastern regions, while lower values are observed in the southwestern and western areas.

### Description of hydrological factors

**Fig 5:**
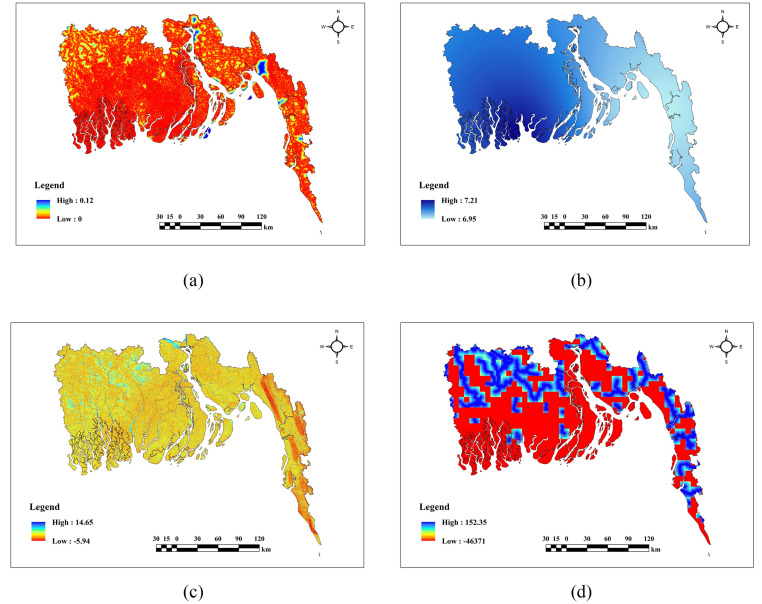
Hydrological factors (a) DtR, (b) MSL, (c) TWI, (d) VDCN.

The hydrological parameters depicted in Fig 5 exhibit diverse spatial distributions throughout the region. DtR(a) and VDCN(d) have elevated values in the northern and western upland regions, while demonstrating lower values in the middle and southeastern regions adjacent to rivers. Mean Sea Level (MSL)(b) reflects higher values in the northwestern and southwestern regions, whereas lower values are observed in the southern and coastal zones, with intermediate levels in the intervening areas. TWI(c) exhibits a comparable pattern, displaying low values in the eastern regions and higher values in the center and low-lying areas in the western regions.

### Description of meteorological factors

**Fig 6:**
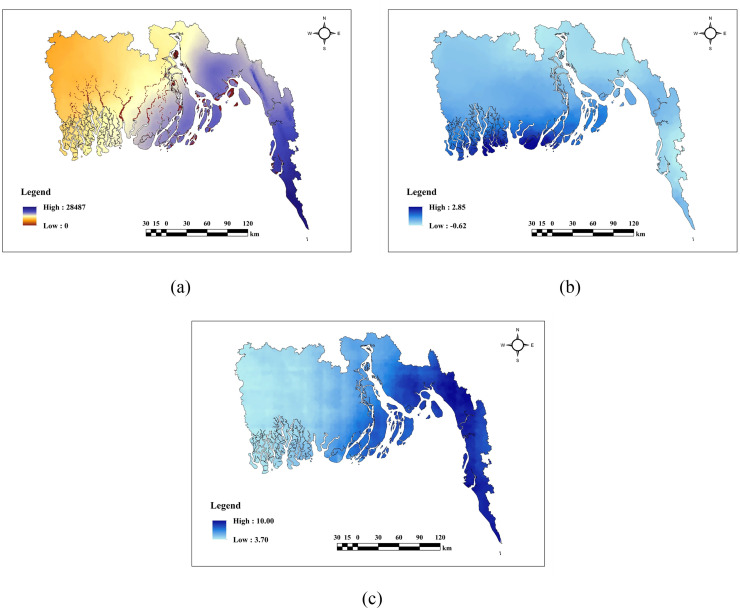
Meteorological factors (a) Aridity Index, (b) Mean Wind Speed, (c) Precipitation.

According to Fig 6 the meteorological factors demonstrate varied regional distributions throughout the region. The Aridity Index(a) and Precipitation(c) exhibit higher levels mostly in the southeastern and northeastern regions, whilst lower values are observed in the southwestern and northwestern sectors. The Mean Wind Speed(b) has a contrasting pattern, with higher values in the southwestern areas and lower values in the eastern parts. Moderate values for all three parameters are typically concentrated in the central areas of the region.

### Description of geological factors

**Fig 7:**
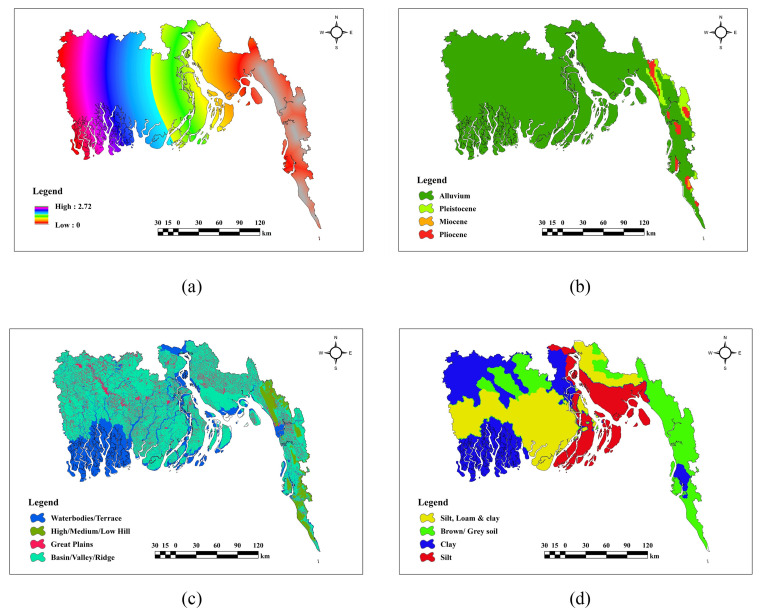
Geological factors (a) DtF, (b) Geology, (c) Geomorphology, (d) Soil Texture.

Fig 7 presents detailed scenarios of geological parameters, including DtF, Geology, Geomorphology, and Soil Texture. Low DtF(a) values are predominantly noted in the east, progressively rising towards the west. The geological map(b) indicates that alluvial predominates in the central and western regions. In terms of Geomorphology(c), prominent high and medium hills, together with ridges, are in the eastern and southeastern regions, while the plains and basins are mostly found in the middle and northern sections. In terms of soil texture(d), silt, loam, and clay soils are prevalent in the center region, whilst brown/gray soils are predominant in the eastern section.

### Description of land use & human activity factors

**Fig 8:**
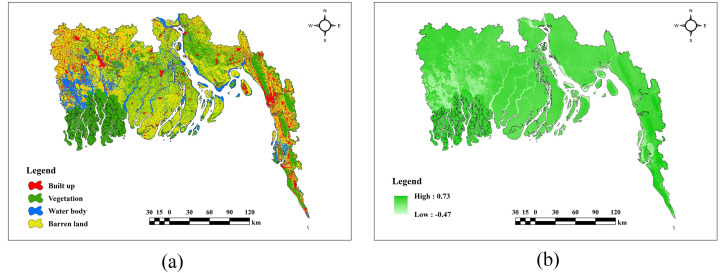
Land use & Human activity factors (a) LULC, (b) NDVI.

In Fig 8, according to LULC(a) map built-up areas are mostly found in the eastern and northwestern regions, whereas vegetation predominates in the southwestern regions. High NDVI(b) values are observed in the eastern and northeastern regions, characterized by extensive vegetation. Moderate NDVI values are allocated between vegetation and urban areas.

### Independent variable selection

**Fig 9:**
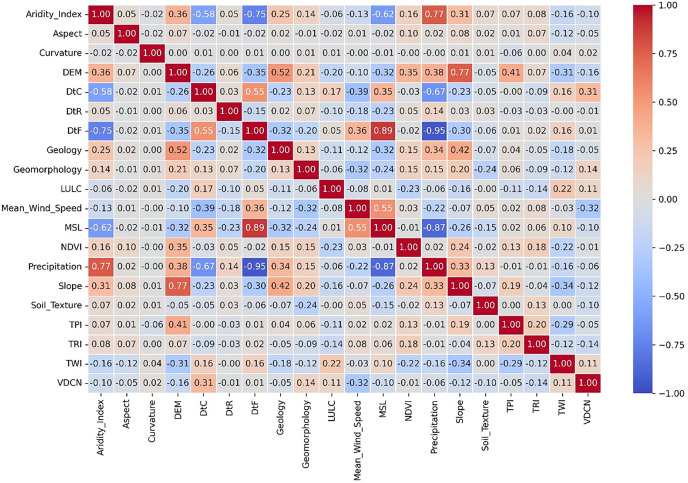
Correlation Matrix of all variables.

Table 3 displays the results of the multicollinearity evaluation and statistical significance testing employed to identify the most impactful factors for the model. Parameters having p-values exceeding 0.05, including average curvature, DtR, LULC, TPI, and TWI, were excluded and denoted with a double star (**). MSL and Precipitation parameters were excluded because of high VIF values, denoted with triple star (***) (>10). The correlation matrix for every variable is shown in Fig 9. The pair MSL and DtF in this matrix exhibited a strong positive correlation (r = 0.89), surpassing the 0.80 threshold for multicollinearity. Consequently, MSL was eliminated from that pair because of its high correlation and excessively high VIF value. Notably, the Aridity Index exhibits a positive correlation with precipitation (r = 0.77), whereas precipitation shows a strong negative correlation with both DtF (r = −0.95) and MSL (r = −0.87). There were also moderate correlations found between DEM and Geology (r = 0.52) and between Slope and DEM (r = 0.77).

**Table 3:** Multicollinearity and Significance Testing for Model Variable Selection.

Feature	VIF	Tolerance	Pearson Correlation	P-Value
Aridity Index	3.48	0.29	0.11	0.001
Aspect	2.97	0.34	−0.03	0.016
Curvature	1.01	0.99	−0.01	0.603**
DEM	6.15	0.16	0.09	0.001
DtC	1.34	0.75	−0.30	0.001
DtR	1.60	0.63	0.01	0.562**
DtF	4.96	0.20	−0.15	0.001
Geology	7.84	0.13	0.10	0.001
Geomorphology	9.26	0.11	−0.16	0.001
LULC	9.06	0.11	0.02	0.389**
Mean Wind Speed	3.13	0.32	0.12	0.001
MSL	459.99***	0.00	−0.13	0.001
NDVI	9.31	0.11	−0.57	0.001
Precipitation	240.81***	0.00	0.22	0.001
Slope	3.74	0.27	0.18	0.001
Soil Texture	7.96	0.13	0.05	0.021
TPI	1.53	0.65	−0.02	0.371**
TRI	2.12	0.47	−0.07	0.001
TWI	1.33	0.75	−0.01	0.779**
VDCN	2.89	0.35	−0.21	0.001

Note: The model excludes variables with p-values above 0.05 (**) and VIFs above 10 (***) because they are statistically insignificant.

**Fig 10:**
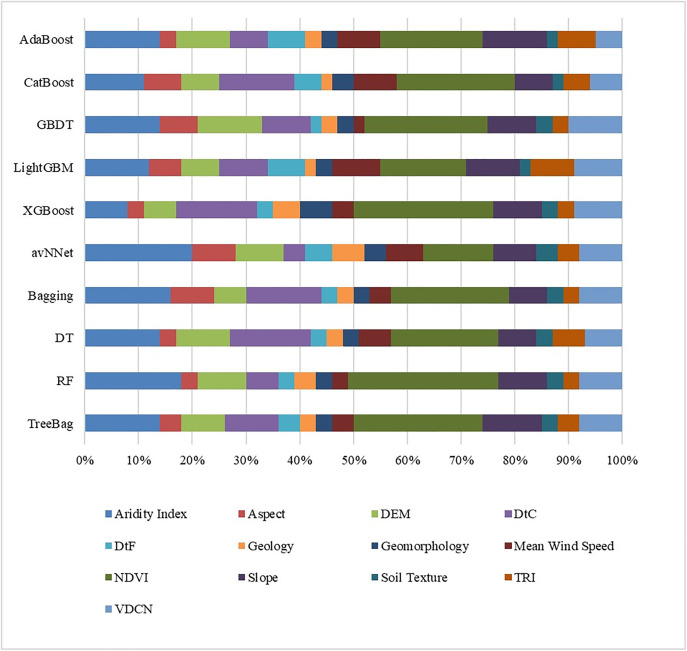
Assessment of the importance of parameters that influence coastal erosion.

Fig 10 indicates that NDVI is the predominant factor in AdaBoost, CatBoost, GBDT, LightGBM, XGBoost, Bagging, DT, RF and TreeBag, with scores of 19, 22, 23, 16, 26, 22, 20, 28 and 24, respectively. Nonetheless, with a value of 20 the Aridity Index is the predominant factor in the avNNet model. The scores of 14, 14, 12, 16, 18 and 14 for the Aridity Index have a significant impact on AdaBoost, GBDT, LightGBM, Bagging, RF, and TreeBag. With scores of 14, 15 and 15 in CatBoost, XGBoost and Decision Trees, respectively, DtC also has a significant influence. Significantly, excluding avNNet, Geology and Soil Texture demonstrate the lowest significance across all models.

### Coastal erosion susceptibility zones distribution based on machine learning models

**Fig 11:**
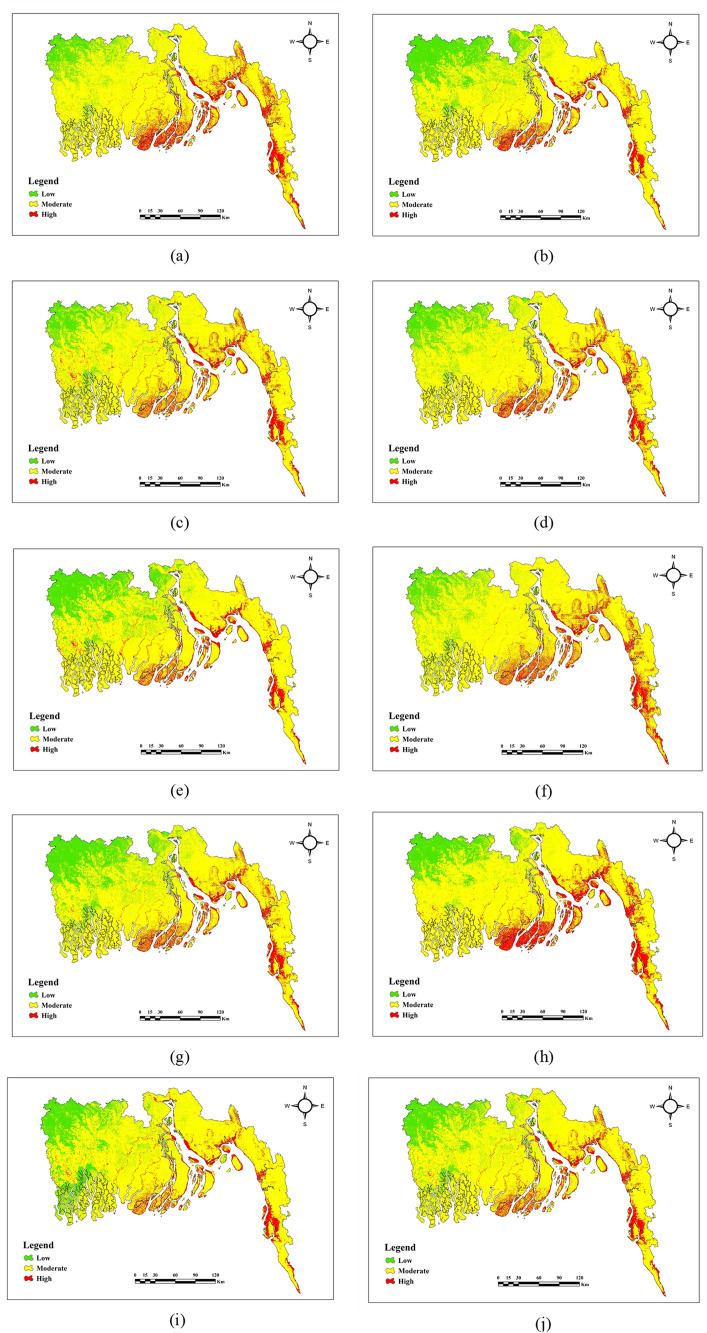
Maps of coastal erosion generated through machine learning methods (a) AdaBoost, (b) CatBoost, (c) GBDT, (d) LightGBM, (e) XGBoost, (f) ANN, (g) Bagging, (h)DT, (i)RF, (j) TreeBag.

Table 4 and Fig 11 illustrate the coastal erosion vulnerability zones categorized into low, moderate, and high impact levels. Table 4 demonstrates that there are comparable patterns among AdaBoost, GBDT, avNNet, and LightGBM, with a high percentage of moderate-impact areas (77.81%–79.36%), low-impact areas (around 10%–12.6%), and high-impact areas (9.58%–12.03%). CatBoost and XGBoost likewise exhibit similar outcomes, with high impact coming in slightly lower at 8.12%–8.81%, low impact areas at about 18%, and moderate impact predominating at 72.68% and 73.58%, respectively. Between 71.82% and 74.36%, the Bagging and DT models have moderate impact; Bagging has the largest low-impact area (19.03%), while DT has a noteworthy high-impact proportion (13.23%). With a moderate impact of almost 76%, RF and TreeBag exhibit comparable patterns; however, RF’s high-impact area is smaller (7.9%) than TreeBag’s (9.04%).

**Table 4:** Coastal Erosion Susceptibility Zones Distribution Based on ML Models.

	Low		Moderate		High	
	Percentage	Area	Percentage	Area	Percentage	Area
AdaBoost	9.28%	4380.12	78.69%	37143.66	12.03%	5676.21
CatBoost	18.50%	8733.79	72.68%	34306.06	8.81%	4160.16
GBDT	10.65%	5025.94	79.36%	37457.76	9.99%	4716.31
LightGBM	12.60%	5948.73	77.81%	36727.76	9.58%	4523.51
XGBoost	18.30%	8636.01	73.58%	34729.97	8.12%	3834.02
avNNet	10.02%	4730.51	79.20%	37381.93	10.78%	5087.56
Bagging	19.03%	8981.01	71.82%	33900.16	9.15%	4318.82
DT	12.41%	5855.37	74.36%	35100.18	13.23%	6244.46
RF	15.96%	7533.13	76.14%	35939.47	7.90%	3727.40
Treebag	14.37%	6784.35	76.59%	36148.45	9.04%	4267.21

### Validation of models by ROC and AUC

**Fig 12:**
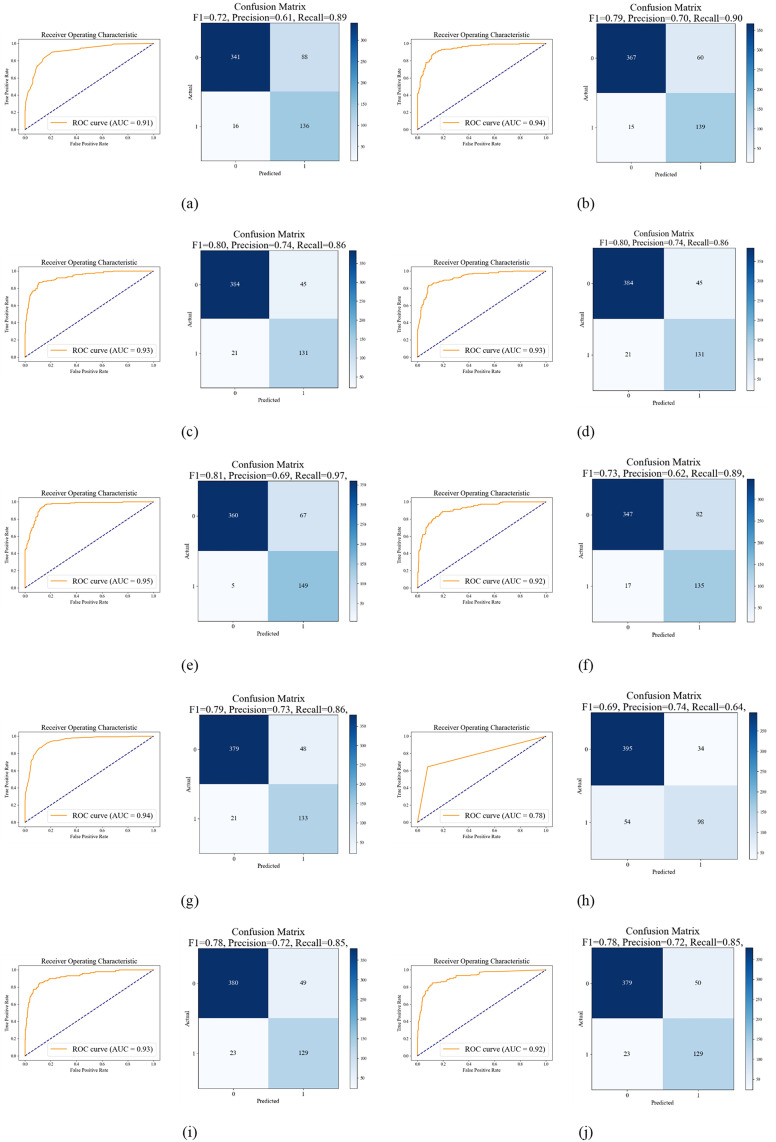
Model validation with Receiver Operating Characteristic (ROC) curve and Confusion matrix for (a) AdaBoost, (b) CatBoost, (c) GBDT, (d) LightGBM, (e) XGBoost, (f) ANN, (g) Bagging, (h)DT, (i)RF, (j) Treebag.

Fig 12 depicts the results of model validation through ROC curves, confusion matrices, F1-scores, precision, and recall, highlighting significant performance disparities. XGBoost proved to be the most effective model, attaining a maximum AUC of 0.95 and an F1-score of 0.81, with precision and recall metrics surpassing 0.68. Close competitors included Bagging and CatBoost, both attaining robust results with AUCs about 0.94 and F1-scores of 0.79. Models including GBDT, Random Forest, and LightGBM demonstrated comparable efficacy, attaining AUCs of 0.93 and F1-scores of 0.78 and 0.80. Both ANN and Treebag demonstrated somewhat suboptimal performance, with AUCs of 0.92 and F1-scores between 0.73 and 0.78. AdaBoost demonstrated marginally inferior performance, achieving an AUC of 0.91 and an F1-score of 0.72. The Decision Tree model had the lowest evaluation, attaining an AUC of 0.78 and an F1-score slightly under 0.70.

### Coastal erosion susceptibility zones distribution by best model among districts

**Fig 13:**
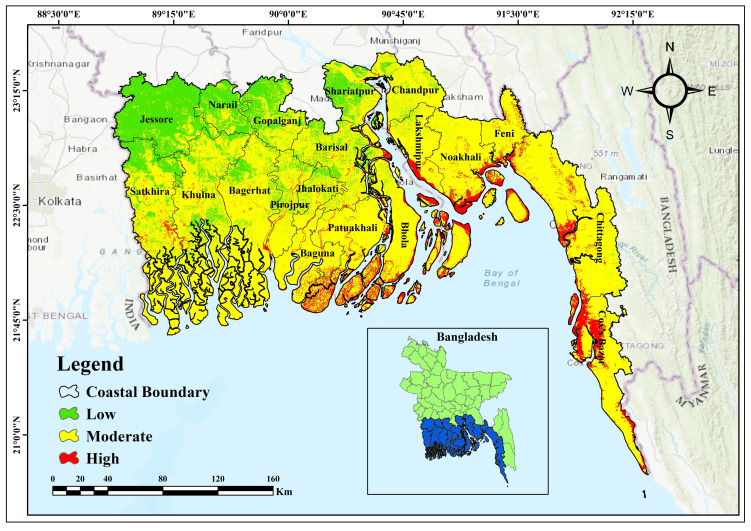
District-wise coastal erosion Susceptibility zones by the best model (XGBoost).

The geographical findings that are derived from the analysis of the XGBoost model presented in Fig 13 and Table 5 reveal that the Jashore and Narail districts possess the highest percentage of areas that are classified as low-risk. The districts take up 86.94% and 71.24% of the respective areas. Shariatpur (46.78%) and Gopalganj (52.71%) are primarily the contributors of this kind of low-risk investment. Districts like Barguna, Chandpur, Feni, and Lakshmipur are predominantly endowed with moderate-risk classes, with a high 91.45% of Barguna’s area falling into this category. Spatial patterns, however, indicate that districts like these are largely dominated by these classes. Bagerhat, Khulna, Pirojpur, and Jhalokati are typical districts with high coverage levels in moderate risks, with all of them exceeding 77%. The spatial pattern reveals that Bhola (19.41%), Cox’s Bazar (26.20%), and Patuakhali (21.47%) have the highest percentage of high-risk areas, thus being the most vulnerable districts. Moreover, districts like Noakhali (16.94%) and Satkhira (3.83%) have significant areas of high-risk areas, which signify their vulnerability in the local risk scenario.

**Table 5:** Coastal erosion Susceptibility zones distribution among districts in the Coastal region by the best model (XGBoost).

	Low		Moderate		High	
	Percentage	Area	Percentage	Area	Percentage	Area
Bagerhat	10.30%	407.78	87.14%	3449.70	2.56%	101.52
Barguna	0.48%	8.72	91.45%	1674.38	8.08%	147.90
Barisal	21.83%	607.85	74.98%	2088.11	3.20%	89.04
Bhola	2.02%	68.70	78.58%	2673.93	19.41%	660.37
Chandpur	13.14%	232.67	85.94%	1397.33	0.91%	15.00
Chittagong	0.83%	43.72	88.40%	4669.95	10.78%	569.33
Cox’s Bazar	0.24%	5.88	73.57%	1833.26	26.20%	652.86
Feni	0.10%	0.98	90.13%	892.26	9.77%	96.76
Gopalganj	52.71%	774.35	47.17%	692.89	0.12%	1.76
Jashore	86.94%	2266.41	12.96%	337.94	0.10%	2.64
Jhalokati	20.27%	143.29	77.91%	550.85	1.82%	12.85
Khulna	18.92%	831.52	78.68%	3457.38	2.39%	105.11
Lakshmipur	4.52%	65.06	85.83%	1235.99	9.65%	138.94
Narail	71.24%	689.62	28.67%	277.49	0.09%	0.88
Noakhali	0.46%	16.98	82.60%	3044.53	16.94%	624.49
Patuakhali	0.60%	19.24	77.93%	2510.19	21.47%	691.57
Pirojpur	16.54%	211.44	80.86%	1033.41	2.59%	33.15
Satkhira	21.43%	817.90	74.74%	2852.90	3.83%	146.20
Shariatpur	46.78%	549.22	52.60%	617.56	0.61%	7.22

### Future climate change scenarios

**Fig 14:**
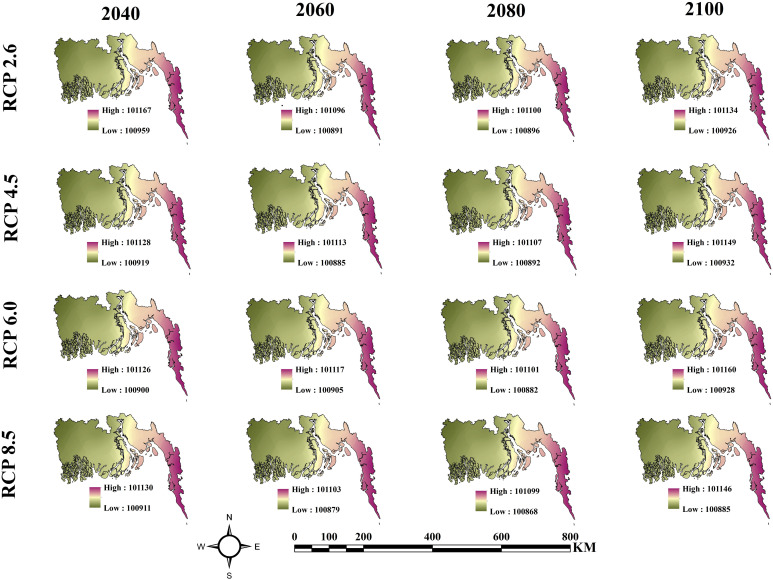
From 2040 to 2100, the annual average sea level pressure for the various RCP scenarios (RCP 2.6, RCP 4.5, RCP 6.0, and RCP 8.5).

According to Fig 14, sea level pressure maps across several RCP scenarios demonstrate that the eastern region exhibits more sea level pressure compared to the western region. In 2040, RCP 2.6 demonstrates a slightly elevated sea level pressure relative to RCP 4.5, 6.0, and 8.5. In 2060, a universal decrease in sea level pressure is noted across all scenarios. By 2080, RCP 8.5 forecasts slightly reduced pressure compared to the other scenarios. By 2100, all Representative Concentration Pathways (RCPs) indicate a little upward trend in sea level pressure.

### Coastal erosion vulnerability zones distribution based on different future climate change scenarios

**Fig 15:**
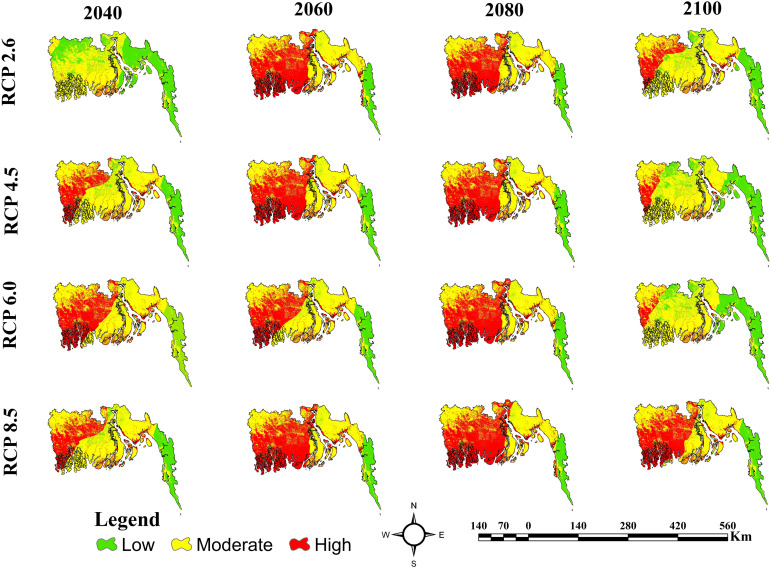
Coastal Erosion Susceptibility for Different RCP Scenarios (RCP 2.6, RCP 4.5, RCP 6.0, and RCP 8.5) from 2040 to 2100 with Best Model (XGBoost).

Table 6 and Fig 15 present coastal erosion Susceptibility zones distributions under various climatic scenarios (RCP 2.6, 4.5, 6.0, and 8.5) for the years 2040, 2060, 2080, and 2100. In 2040, RCP 2.6 is characterized by a significant Low category (42%) and a minimal High category (3%), but RCP 4.5, 6.0, and 8.5 exhibit diverse distributions, with RCP 6.0 presenting the most substantial High category (35%). By 2060, RCP 2.6 and 8.5 indicate a substantial transition, with around 47% of the area classified as High, whilst RCP 4.5 and 6.0 also demonstrate an increasing trend in the High category; however, RCP 6.0 exhibits a more equitable distribution (13% Low, 53% Moderate, 34% High). By 2080, a comparable trend persists, with RCP 6.0 and 8.5 exhibiting the highest High category percentages at 48% and 50%, respectively, and RCP 2.6 and 4.5 are at 44% and 45%. By 2100, RCP 2.6, 4.5, and 6.0 exhibit a marked decrease in the High category (between 8% and 19%) and an increase in the Low category, especially in RCP 6.0 (31%). Simultaneously, RCP 8.5 with 40% of the area is still designated as High.

**Table 6:** Coastal erosion Susceptibility zones distribution according to Different RCP Scenarios (RCP 2.6, RCP 4.5, RCP 6.0, and RCP 8.5) from 2040 to 2100 with Best Model.

			Low	Moderate	High
2040	RCP 2.6	%	42%	55%	3%
		Area	19906.75	25759.69	1533.564
	RCP 4.5	%	16%	60%	24%
		Area	7431.633	28403.71	11364.66
	RCP 6.0	%	13%	52%	35%
		Area	6105.21	24414.56	16680.23
	RCP 8.5	%	15%	59%	26%
		Area	7039.616	27928.71	12231.68
2060	RCP 2.6	%	9%	44%	47%
		Area	4268.691	20670.52	22260.79
	RCP 4.5	%	11%	44%	45%
		Area	5121.893	20803.06	21275.04
	RCP 6.0	%	13%	53%	34%
		Area	5980.069	25211.75	16008.18
	RCP 8.5	%	10%	43%	47%
		Area	4653.039	20262.8	22284.16
2080	RCP 2.6	%	10%	45%	44%
		Area	4836.109	21417.6	20946.29
	RCP 4.5	%	11%	45%	45%
		Area	5121.866	21024.81	21053.32
	RCP 6.0	%	9%	43%	48%
		Area	4418.093	20203.93	22577.97
	RCP 8.5	%	9%	41%	50%
		Area	4146.771	19279.35	23773.88
2100	RCP 2.6	%	19%	63%	19%
		Area	8842.319	29529	8828.68
	RCP 4.5	%	28%	61%	11%
		Area	13215.88	28961.58	5022.535
	RCP 6.0	%	31%	61%	8%
		Area	14515.5	28688.74	3995.765
	RCP 8.5	%	13%	47%	40%
		Area	6250.205	22178.32	18771.48

## Discussion

This study analytically evaluated coastal erosion susceptibility in Bangladesh using sophisticated machine learning algorithms that combined topographical, hydrological, climatic, geological, and human elements. The Normalized Difference Vegetation Index (NDVI) was identified as the most significant variable by feature selection procedures, specifically Pearson correlation, variance inflation factor (VIF), and p-values. The significance of vegetation cover in reducing the danger of erosion was highlighted by the NDVI with up to 28% contribution in the Random Forest (RF) model and 26% in XGBoost. Another important component was the Aridity Index, which made up 20% of avNNet and 12–18% of ensemble configurations. With contributions of 15% in XGBoost and 14–16% in other models, Distance to Coast (DtC) continuously placed highly across all models. On the other hand, geological factors including soil texture and geology had lower significance ratings, usually less than 6%, suggesting that they had little bearing on the variability of erosion in space. These results are reliable with earlier research by R. Bera and R. Maiti [137] and Islam et al. [138], who also highlighted the supremacy of NDVI and coastal proximity in determining coastal susceptibility. The results, however, are in contrast to those of Gao et al. [139], whose modeling approach based on RUSLE gave geomorphological variables more weight. The deterministic nature of RUSLE models, which lack the adaptive learning capability found in the machine learning approaches employed here, is probably the cause of the discrepancy.

Ensemble machine learning methods consistently beat individual classifiers in terms of model performance. With an area under the curve (AUC) score of 0.95, XGBoost outperformed CatBoost and Bagging, which were closely followed by GBDT, RF, and LightGBM (0.93). With an AUC of only 0.78, simpler models such as Decision Tree performed poorly. High-risk zones ranged from 7.9% (RF) to 13.23% (DT), whereas moderate-risk zones, which ranged from 71.82% (Bagging) to 79.36% (GBDT), spanned the largest areas across all models. Particularly, the AUC values in this study surpass those reported by Islam et al. [138] for distinctive between high and low erosion susceptibility zones in the Chattogram coastal region, excellent model performance is indicated by the AUC value of 0.909. Additionally, Bammou et al. [140] documented an AUC of 0.96 for the XGBoost model in their soil erosion evaluation, corroborating the dependability of superior performance results in analogous situations. This suggests that the existing ensemble configurations for coastal erosion scenarios have a greater level of predictive dependability. A different result was reported by Rahmati et al. [141], for susceptibility mapping in Iran, Random Forest (AUC 0.91) performed somewhat better than XGBoost (AUC 0.89). This demonstrates how dataset properties and regional conditions can affect model performance.

According to the high-resolution susceptibility maps created for this study, Bhola (19.41%), Cox’s Bazar (26.20%), and Patuakhali (21.47%) are some of the districts that are most susceptible to erosion at the moment. Conversely, districts with large percentages of low-risk zones include Jashore (86.94%), Narail (71.24%), and Gopalganj (52.71%). This is probably because of things like higher elevation, more vegetation, and a more inland location. Many districts, such as Barguna (91.45%), and Feni (90.13%), are dominated by moderate-risk zones. The spatial distribution of erosion susceptibility resembles well with [142,143] who also recognized Patuakhali and Cox’s Bazar as areas for vulnerability. Although their research focused on tidal-driven riverbank erosion, the current findings point to a combined effect of low elevation, vegetation loss, and weather stresses. Anthropogenic and climatic factors, which are frequently missing from deterministic models like GloSEM and RUSLE, allow for this wider viewpoint. This makes the model a more complete tool for both current and future coastal erosion risk assessment.

This research is distinct from previous studies that it combines CMIP5-based climate scenarios. The risk of coastal erosion is predicted to significantly increase under future climate scenarios, especially under high-emission pathways. High-risk areas are expected to rise from 26% in 2040 to a peak of 50% by 2080, then slightly fall to 40% by 2100, according to RCP 8.5. RCP 6.0 shows similar patterns, with high-risk zones reaching a peak of 48% by 2080. Peak high-risk area coverage, on the other hand, rises more moderately at RCP 2.6 and 4.5, reaching 47% and 45%, respectively. These forecasts highlight how severe coastal damage could occur in the event of unchecked climate change. These patterns align with the work of [144], who documented faster coastline retreat in high sea-level rise scenarios. However, compared to the regional-scale estimates seen in previous work, the spatially explicit outputs of this analysis provide a more detailed view of risk distribution, increasing their usefulness for local adaptation planning.

By combining machine learning, climate modeling, and geospatial analysis, this study creates a solid, data-driven framework for evaluating the danger of coastal erosion. The models are useful tools for predicting erosion under present and future conditions because of their excellent predictive ability and good spatial resolution. This hybrid methodology provides more generalizability and actionable insights for resilient coastal management and governance as compared to traditional approaches used in coastal Bangladesh. Although the modeling framework employed in this study is technically transferable to other geographic locations, its predictability and interpretability will necessarily be region-dependent. Geomorphological variation including coral reefs, deltas, cliffs, and climatic regimes like monsoonal versus arid regimes, and land use necessitate extensive recalibration of the model using local input variables to yield significant and credible results. Not only this but also the lack of real-time erosion monitoring could compromise temporal accuracy. Future research should focus on the transfer and calibration of this framework across different geographic and climatic settings, employing local parameters, and also real-time monitoring to enhance model robustness, generalizability, and applicability across different coastal systems.

## Conclusion

The security of Bangladesh’s shoreline is in danger because sea levels are rising faster, climate change is getting worse, and the land is becoming less stable. Using modern machine learning methods and 20 important meteorological, geographical, hydrological, tropological, and land-use factors, this study looked at how Susceptibility the coast is to erosion. The main things that affect erosion risk are the normalized difference vegetation index (NDVI). To find areas that are likely to erode, different ensemble learning methods were used, such as bagging (Random Forest, Bagging, Decision Tree, Treebag, averaging Neural Network) and boosting (XGBoost, LightGBM, CatBoost, GBDT, AdaBoost). XGBoost was used to get an area under the curve (AUC) of 0.95, which means it was better at predicting the future and finding high-risk areas. The results show big differences in geography, with most of the high-risk places being in Bhola, Cox’s Bazar, and Patuakhali. This was the breakdown of high-risk places in these three areas: 19.41%, 26.20%, and 21.47%. Barguna (91.45%), and Feni (90.13%) have a relatively high number of places that are considered to have moderate risk. RCP models 2.6, 4.5, 6.0, and 8.5 all predict that the number of high-risk areas will grow in the years 2040, 2060, 2080, and 2100. This is especially true for RCP 8.5, which says that shares will grow by 50% by 2080 and by 40% by 2100. When the RCP number is 2.6, 4.5, or 6.0, it means that the risk has changed, though only slightly. These results show how important it is to build buildings that can withstand climate change, use methods to stop erosion, and make policies that adapt to new situations. It is important to protect especially Susceptibility groups by setting up map early warning systems, enforcing zoning laws, building marine embankments, and planting trees. Our results also point to the ethical challenge of coastal erosion management, since a lot of the high-risk zones coincide with marginalized groups. Policymakers must utilize these projections to create inclusive plans that reduce displacement risks and promote equitable access to resources. Incorporating community-based adaptation and livelihood assistance will be crucial for equitable and sustainable coastal resilience planning. Even though this study is very thorough, it has some limitations. If scholars do not take into account changing socio-economic factors like changes in land use and the building of new infrastructure, the risk of erosion might be underrated. Mitigation strategies such as population relocation can also disproportionately impact underprivileged people, resulting in loss of livelihoods, displacement, or limited access to critical services. Therefore, evaluations of erosion risk and policy measures must take into account socioeconomic and environmental factors in order to achieve inclusive and equitable results. To enhance predictive accuracy, future studies ought to make use of integrated socio-environmental databases, real-time monitoring, and high-resolution remote sensing. The UN’s Sustainable Development Goals (SDG) 11 (Sustainable Cities and Communities) and 13 (Climate Action) are in line with this work. It gives useful information that could help with managing the coast and making plans for long-term protection in Bangladeshi places that are prone to erosion.
